# Supplementary material for: Identification Of Protein Cargo in Extracellular Vesicles from Macrophages in Progressing and Regressing Tumors
Source: bioRxiv. 2025 Nov 6:2025.11.05.685429. Preprint. [Version 1] doi: 10.1101/2025.11.05.685429 (PMC12637521; doi:10.1101/2025.11.05.685429)
Supplement: Supplement 4 [file media-4.pdf]

Supplemental Table 1

M1 BMDM-EV signature proteins

From	Entry	Reviewed	Entry Name	Protein names	Gene Names	Organism	Length
P04918	P04918	reviewed	SAA3_MOUSE	Serum amyloid A-3 protein	Saa3	Mus musculus (Mouse)	122
P17182	P17182	reviewed	ENOA_MOUSE	Alpha-enolase (EC 4.2.1.11) (2-phospho-D-glycerate hydro-lyase) (Enolase 1) (Non-neural enolase) (NNE)	Eno1 Eno-1	Mus musculus (Mouse)	434
Q07797	Q07797	reviewed	LG3BP_MOUSE	Galectin-3-binding protein (Cyp-C-associated protein) (CyCAP) (Lectin galactoside-binding soluble 3-binding protein) (Protein MAMA)	Lgals3bp Cycap Mama	Mus musculus (Mouse)	577
Q61207	Q61207	reviewed	SAP_MOUSE	Prosaposin (Sulfated glycoprotein 1) (SGP-1) [Cleaved into: Saposin-A; Saposin-B-Val; Saposin-B; Saposin-C; Saposin-D]	Psap Sgp1	Mus musculus (Mouse)	557
A0A0U1RNR0	A0A0U1RNR0	unreviewed	A0A0U1RNR0_MOUSE	Hormone-sensitive lipase (EC 3.1.1.23) (EC 3.1.1.79) (Monoacylglycerol lipase LIPE) (Retinyl ester hydrolase)	Lipe	Mus musculus (Mouse)	180
P01027	P01027	reviewed	CO3_MOUSE	Complement C3 (HSE-MSF) [Cleaved into: Complement C3 beta chain; C3-beta-c (C3bc); Complement C3 alpha chain; C3a anaphylatoxin; Acylation stimulating protein (ASP) (C3adesArg); Complement C3b (Complement C3b-alpha' chain); Compler	C3	Mus musculus (Mouse)	1663
P09528	P09528	reviewed	FRIH_MOUSE	Ferritin heavy chain (Ferritin H subunit) (EC 1.16.3.1) [Cleaved into: Ferritin heavy chain, N-terminally processed]	Fth1 Fth	Mus musculus (Mouse)	182
P09581	P09581	reviewed	CSF1R_MOUSE	Macrophage colony-stimulating factor 1 receptor (CSF-1 receptor) (CSF-1-R) (CSF-1R) (M-CSF-R) (EC 2.7.10.1) (Proto-oncogene c-Fms) (CD antigen CD115)	Csf1r Csfmr Fms	Mus musculus (Mouse)	977
P14106	P14106	reviewed	C1QB_MOUSE	Complement C1q subcomponent subunit B	C1qb	Mus musculus (Mouse)	253
P98086	P98086	reviewed	C1QA_MOUSE	Complement C1q subcomponent subunit A	C1qa	Mus musculus (Mouse)	245
P98203	P98203	reviewed	ARVC_MOUSE	Splicing regulator ARVCF (Armadillo repeat protein deleted in velo-cardio-facial syndrome homolog)	Arvcf	Mus musculus (Mouse)	962
Q60963	Q60963	reviewed	PAFA_MOUSE	Platelet-activating factor acetylhydrolase (PAF acetylhydrolase) (EC 3.1.1.47) (1-alkyl-2-acetylglycerophosphocholine esterase) (2-acetyl-1-alkylglycerophosphocholine esterase) (LDL-associated phospholipase A2) (LDL-PLA(2)) (PAF 2-acylhydrolase)	Pla2g7 Pafah	Mus musculus (Mouse)	440
Q62266	Q62266	reviewed	SPR1A_MOUSE	Cornifin-A (Small proline-rich protein 1A) (SPR1 A) (SPR1A)	Sprr1a	Mus musculus (Mouse)	144
E9Q557	E9Q557	reviewed	DESP_MOUSE	Desmoplakin (DP)	Dsp	Mus musculus (Mouse)	2883
P10639	P10639	reviewed	THIO_MOUSE	Thioredoxin (Trx) (ATL-derived factor) (ADF)	Txn Txn1	Mus musculus (Mouse)	105
P17742	P17742	reviewed	PPIA_MOUSE	Peptidyl-prolyl cis-trans isomerase A (PPIase A) (EC 5.2.1.8) (Cyclophilin A) (Cyclosporin A-binding protein) (Rotamase A) (SP18) [Cleaved into: Peptidyl-prolyl cis-trans isomerase A, N-terminally processed]	Ppia	Mus musculus (Mouse)	164
Q02257	Q02257	reviewed	PLAK_MOUSE	Junction plakoglobin (Desmoplakin III) (Desmoplakin-3)	Jup	Mus musculus (Mouse)	745

M2 BMDM-EV signature proteins

From	Entry	Reviewed	Entry Name	Protein names	Gene Names	Organism	Length
P01756	P01756	reviewed	HVM12_MOUSE	Ig heavy chain V region MOPC 104E		Mus musculus (Mouse)	117
P05064	P05064	reviewed	ALDOA_MOUSE	Fructose-bisphosphate aldolase A (EC 4.1.2.13) (Aldolase 1) (Muscle-type aldolase)	Aldoa Aldo1	Mus musculus (Mouse)	364
P16110	P16110	reviewed	LEG3_MOUSE	Galectin-3 (Gal-3) (35 kDa lectin) (Carbohydrate-binding protein 35) (CBP 35) (Galactose-specific lectin 3) (IgE-binding protein) (L-34 galactoside-binding lectin) (Laminin-binding protein) (Lectin L-29) (Mac-2 antigen)	Lgals3	Mus musculus (Mouse)	264
P62242	P62242	reviewed	RS8_MOUSE	Small ribosomal subunit protein eS8 (40S ribosomal protein S8)	Rps8	Mus musculus (Mouse)	208
P62806	P62806	reviewed	H4_MOUSE	Histone H4	H4c1 Hist1h4a; H4c2 H4-53 Hist1h4b; H4c3 H4-12 Hist1h4c;	Mus musculus (Mouse)	103
Q6GQT1	Q6GQT1	reviewed	A2MG_MOUSE	Alpha-2-macroglobulin-P (Alpha-2-macroglobulin)	A2m A2mp	Mus musculus (Mouse)	1474
O35744	O35744	reviewed	CHIL3_MOUSE	Chitinase-like protein 3 (EC 3.2.1.52) (Beta-N-acetylhexosaminidase Ym1) (Chitinase-3-like protein 3) (ECF-L) (Eosinophil chemotactic cytokine) (Secreted protein Ym1)	Chil3 Chi3l3 Ym1	Mus musculus (Mouse)	398
O70435	O70435	reviewed	PSA3_MOUSE	Proteasome subunit alpha type-3 (Macropain subunit C8) (Multicatalytic endopeptidase complex subunit C8) (Proteasome component C8) (Proteasome subunit K) (Proteasome subunit alpha-7) (alpha-7)	Psma3	Mus musculus (Mouse)	255
O88342	O88342	reviewed	WDR1_MOUSE	WD repeat-containing protein 1 (Actin-interacting protein 1) (AIP1)	Wdr1	Mus musculus (Mouse)	606
P04117	P04117	reviewed	FABP4_MOUSE	Fatty acid-binding protein, adipocyte (3T3-L1 lipid-binding protein) (Adipocyte lipid-binding protein) (ALBP) (Adipocyte-type fatty acid-binding protein) (A-FABP) (AFABP) (Fatty acid-binding protein 4) (Myelin P2 protein homolog) (P15) (P2 adipocyte protein) (Protein 422)	Fabp4 Ap2	Mus musculus (Mouse)	132
P09066	P09066	reviewed	HME2_MOUSE	Homeobox protein engrailed-2 (Homeobox protein en-2) (Mo-En-2)	En2 En-2	Mus musculus (Mouse)	324
P10107	P10107	reviewed	ANXA1_MOUSE	Annexin A1 (Annexin I) (Annexin-1) (Calpactin II) (Calpactin-2) (Chromobindin-9) (Lipocortin I) (Phospholipase A2 inhibitory protein) (p35) [Cleaved into: Annexin Ac2-26]	Anxa1 Anx1 Lpc-1 Lpc1	Mus musculus (Mouse)	346
P12265	P12265	reviewed	BGLR_MOUSE	Beta-glucuronidase (EC 3.2.1.31)	Gusb Gus Gus-s	Mus musculus (Mouse)	648
P13020	P13020	reviewed	GELS_MOUSE	Gelsolin (Actin-depolymerizing factor) (ADF) (Brevin)	Gsn Gsb	Mus musculus (Mouse)	780
P14069	P14069	reviewed	S10A6_MOUSE	Protein S100-A6 (5B10) (Calcyclin) (Prolactin receptor-associated protein) (S100 calcium-binding protein A6)	S100a6 Cacy	Mus musculus (Mouse)	89
P16045	P16045	reviewed	LEG1_MOUSE	Galectin-1 (Gal-1) (14 kDa lectin) (Beta-galactoside-binding lectin L-14-I) (Galaptin) (Lactose-binding lectin 1) (Lectin galactoside-binding soluble 1) (S-Lac lectin 1)	Lgals1 Gbp	Mus musculus (Mouse)	135
P17742	P17742	reviewed	PPIA_MOUSE	Peptidyl-prolyl cis-trans isomerase A (PPIase A) (EC 5.2.1.8) (Cyclophilin A) (Cyclosporin A-binding protein) (Rotamase A) (SP18) [Cleaved into: Peptidyl-prolyl cis-trans isomerase A, N-terminally processed]	Ppia	Mus musculus (Mouse)	164
P20060	P20060	reviewed	HEXB_MOUSE	Beta-hexosaminidase subunit beta (EC 3.2.1.52) (Beta-N-acetylhexosaminidase subunit beta) (Hexosaminidase subunit B) (N-acetyl-beta-glucosaminidase subunit beta)	Hexb	Mus musculus (Mouse)	536
P20152	P20152	reviewed	VIME_MOUSE	Vimentin	Vim	Mus musculus (Mouse)	466
P26041	P26041	reviewed	MOES_MOUSE	Moesin (Membrane-organizing extension spike protein)	Msn	Mus musculus (Mouse)	577
P40124	P40124	reviewed	CAP1_MOUSE	Adenylyl cyclase-associated protein 1 (CAP 1)	Cap1 Cap	Mus musculus (Mouse)	474
P40142	P40142	reviewed	TKT_MOUSE	Transketolase (TK) (EC 2.2.1.1) (P68)	Tkt	Mus musculus (Mouse)	623
P48036	P48036	reviewed	ANXA5_MOUSE	Annexin A5 (Anchorin CII) (Annexin V) (Annexin-5) (Calphobindin I) (CPB-I) (Endonexin II) (Lipocortin V) (Placental anticoagulant protein 4) (PP4) (Placental anticoagulant protein I) (PAP-I) (Thromboplastin inhibitor) (Vascular anticoagulant-alpha) (VAC-alpha)	Anxa5 Anx5	Mus musculus (Mouse)	319
P48678	P48678	reviewed	LMNA_MOUSE	Prelamin-A/C [Cleaved into: Lamin-A/C]	Lmna Lmn1	Mus musculus (Mouse)	665
P61982	P61982	reviewed	1433G_MOUSE	14-3-3 protein gamma [Cleaved into: 14-3-3 protein gamma, N-terminally processed]	Ywhag	Mus musculus (Mouse)	247
P62814	P62814	reviewed	VATB2_MOUSE	V-type proton ATPase subunit B, brain isoform (V-ATPase subunit B 2) (Endomembrane proton pump 58 kDa subunit) (Vacuolar proton pump subunit B 2)	Atp6v1b2 Atp6b2 Vat2	Mus musculus (Mouse)	511
P62897	P62897	reviewed	CYC_MOUSE	Cytochrome c, somatic	Cycs	Mus musculus (Mouse)	105
P62962	P62962	reviewed	PROF1_MOUSE	Profilin-1 (Profilin I)	Pfn1	Mus musculus (Mouse)	140
Q01853	Q01853	reviewed	TERA_MOUSE	Transitional endoplasmic reticulum ATPase (TER ATPase) (EC 3.6.4.6) (15S Mg(2+)-ATPase p97 subunit) (Valosin-containing protein) (VCP)	Vcp	Mus musculus (Mouse)	806
Q08857	Q08857	reviewed	CD36_MOUSE	Platelet glycoprotein 4 (Glycoprotein IIIb) (GPIIIB) (PAS IV) (PAS-4) (Platelet glycoprotein IV) (GPIV) (CD antigen CD36)	Cd36	Mus musculus (Mouse)	472
Q60692	Q60692	reviewed	PSB6_MOUSE	Proteasome subunit beta type-6 (EC 3.4.25.1) (Low molecular mass protein 19) (Macropain delta chain) (Multicatalytic endopeptidase complex delta chain) (Proteasome delta chain) (Proteasome subunit Y) (Proteasome subunit beta-1) (beta-1)	Psmb6 Lmp19	Mus musculus (Mouse)	238
Q61171	Q61171	reviewed	PRDX2_MOUSE	Peroxiredoxin-2 (EC 1.11.1.24) (Thiol-specific antioxidant protein) (TSA) (Thioredoxin peroxidase 1) (Thioredoxin-dependent peroxide reductase 1) (Thioredoxin-dependent peroxiredoxin 2)	Prdx2 Tdpx1 Tpx	Mus musculus (Mouse)	198
E9PVG8	E9PVG8	unreviewed	E9PVG8_MOUSE	Mucin-2	Fcgbpl1 9530053A07Rik	Mus musculus (Mouse)	2581
O70370	O70370	reviewed	CATS_MOUSE	Cathepsin S (EC 3.4.22.27)	Ctss Cats	Mus musculus (Mouse)	340
P01837	P01837	reviewed	IGKC_MOUSE	Immunoglobulin kappa constant (Ig kappa chain C region MOPC 21)	Igkc	Mus musculus (Mouse)	107
P01942	P01942	reviewed	HBA_MOUSE	Hemoglobin subunit alpha (Alpha-globin) (Hemoglobin alpha chain) [Cleaved into: Hemopressin]	Hba Hba-a1	Mus musculus (Mouse)	142
P02088	P02088	reviewed	HBB1_MOUSE	Hemoglobin subunit beta-1 (Beta-1-globin) (Hemoglobin beta-1 chain) (Hemoglobin beta-major chain)	Hbb-b1	Mus musculus (Mouse)	147
P07724	P07724	reviewed	ALBU_MOUSE	Albumin	Alb Alb-1 Alb1	Mus musculus (Mouse)	608
P08905	P08905	reviewed	LYZ2_MOUSE	Lysozyme C-2 (EC 3.2.1.17) (1,4-beta-N-acetylmuramidase C) (Lysozyme C type M)	Lyz2 Lyz Lyzs	Mus musculus (Mouse)	148
P11680	P11680	reviewed	PROP_MOUSE	Properdin (Complement factor P)	Cfp Pfc	Mus musculus (Mouse)	464
P21460	P21460	reviewed	CYTC_MOUSE	Cystatin-C (Cystatin-3)	Cst3	Mus musculus (Mouse)	140
P29391	P29391	reviewed	FRIL1_MOUSE	Ferritin light chain 1 (Ferritin L subunit 1)	Ftl1 Ftl Ftl-1	Mus musculus (Mouse)	183

Supplemental Table 1. Signature EV protein identities for M1 and M2 BMDMs. The workflow is reflected by Supplemental Figure 3.
